# Conditioning the microenvironment for soft tissue regeneration in a cell free scaffold

**DOI:** 10.1038/s41598-021-92732-9

**Published:** 2021-06-25

**Authors:** Irini Gerges, Margherita Tamplenizza, Federico Martello, Stefano Koman, Giulia Chincarini, Camilla Recordati, Mariacaterina Tamplenizza, Scott Guelcher, Maurizio Crestani, Alessandro Tocchio

**Affiliations:** 1 Tensive SRL, Via Timavo 34, Milan, Italy; 2 Dipartimento di Medicina Veterinaria, Università Degli Studi di Milano, Via dell’Università, 6, 26900 Lodi, Italy; 3 Department of Chemical and Biomolecular Engineering, Vanderbilt University, 2301 Vanderbilt Place, PMB 351604, Nashville, TN 37235-1604 USA; 4 Dipartimento di Scienze Farmacologiche e Biomolecolari – DiSFeB, Università Degli Studi Di Milano, Via Balzaretti, 9, 20133 Milan, Italy

**Keywords:** Biomaterials, Regenerative medicine, Tissue engineering, Health care

## Abstract

The use of cell-free scaffolds for the regeneration of clinically relevant volumes of soft tissue has been challenged, particularly in the case of synthetic biomaterials, by the difficulty of reconciling the manufacturing and biological performance requirements. Here, we investigated in vivo the importance of biomechanical and biochemical cues for conditioning the 3D regenerative microenvironment towards soft tissue formation. In particular, we evaluated the adipogenesis changes related to 3D mechanical properties by creating a gradient of 3D microenvironments with different stiffnesses using 3D Poly(Urethane-Ester-ether) PUEt scaffolds. Our results showed a significant increase in adipose tissue proportions while decreasing the stiffness of the 3D mechanical microenvironment. This mechanical conditioning effect was also compared with biochemical manipulation by loading extracellular matrices (ECMs) with a PPAR-γ activating molecule. Notably, results showed mechanical and biochemical conditioning equivalency in promoting adipose tissue formation in the conditions tested, suggesting that adequate mechanical signaling could be sufficient to boost adipogenesis by influencing tissue remodeling. Overall, this work could open a new avenue in the design of synthetic 3D scaffolds for microenvironment conditioning towards the regeneration of large volumes of soft and adipose tissue, with practical and direct implications in reconstructive and cosmetic surgery.

## Introduction

Current soft tissue reconstruction approaches depend on inert fillers or autologous grafts to replace the lost volume. Despite the clinical success of these techniques, patients suffer from multiple drawbacks such as donor-site morbidity and volume loss over time. Therefore, an unmet clinical need still exists for efficacious solutions for soft tissue restoration after trauma or surgical removal of lesioned tissues^1,2^. In particular, breast cancer reconstructive treatments, which have one of medicine's highest reoperation rates, would greatly benefit from a successful regeneration of relevant soft tissue volumes. In this context, cell-free scaffold-based approaches are emerging as a promising solution due to their biocompatibility, properties modulation/adaptability to that of the target tissue, cost-effectiveness^3,4^, and compliance with international manufacturing standards (es. ISO 13,485, EU 2017/745 Medical Device Regulation and cGMP regulations for USA)^5–7^. Moreover, synthetic scaffolds also represent a more scalable solution in clinics because they can avoid regulatory and manufacturing hurdles typical of the cell-based therapies^8^.

In the last decade, 3D scaffolds for the regeneration of clinically relevant soft tissue volumes have seen considerable progress, especially at the preclinical level, as exemplified by manufacturing of poly(D,L)-lactide and medical grade polycaprolactone scaffolds by fused deposition modelling^1,9^. However, their use is challenged by the high local stiffness of the polymer filaments which fails to match that of the target tissue despite reductions in the filament thickness to a few hundred microns. This is not surprising since the adipose tissue regeneration process is one of the most challenging among connective tissues due to its structural and mechanical complexity and sensitivity to signals, such as the ones from hormone and nervous systems^10,11^. Therefore, developing scaffolds for adipose tissue regeneration required control over biomechanical cues to achieve appropriate cell/biomaterial interactions, which is fundamental to drive the differentiation of mesenchymal stem cells towards adipogenesis^12^.

The effect of mechanical cues on human adipocyte function was previously demonstrated in vitro by Pellegrinelli et. al.^13^. In Young et. al., adult adipose-derived stem cells cultured on soft hydrogels that mimicked the stiffness of adipose tissue (2 kPa) showed significant upregulation of adipogenic markers in vitro^14^. However, in a more physiologically relevant 3D in vivo scenario, the evaluation of the impact of mechanical cues on adipogenesis is more complex, mainly due to the interdependence of cell viability and differentiation with the foreign body response^15^.

In our previous studies, we addressed key factors impacting the biological performance of polyurethane-based crosslinked porous biomaterials as scaffolds for soft tissue regeneration, focusing the attention on the role of polymer chemistry and the microarchitecture^16–18^. These findings enabled our group to develop 3D scaffolds with physicochemical and morphological properties guiding cell infiltration to the scaffold core and rapid recruitment of vascular tissue. In the present work, by modifying the composition of polyester triol segments co-polymerized in the polyurethane network, we synthetized a gradient of Poly(Urethane-Ester-ether) PUEt porous scaffolds sharing similar physicochemical and morphological properties but displaying different substrate stiffnesses. This experimental configuration allowed us to investigate the effects of mechanical cues on adipogenesis. We also compared the effects of mechanical and biochemical conditioning on the regenerative microenvironment using a PUEt scaffold loaded with a peroxisome proliferator–activated receptor γ (PPAR- γ) agonist (Rosiglitazone, RG) for the induction of adipocyte differentiation. The effects of PPAR- γ-loaded scaffolds were compared with unloaded control. The findings from this study are discussed in view of the emerging trends for developing implantable devices for adipose tissue regeneration with particular emphasis on breast reconstruction surgeries.

## Results

### Part I: The impact of biomechanical conditioning on the biological performance in vivo

#### Tuning the PUEt scaffold stiffness as a function of the ratio CL: Gly in the polyester: rationale of the synthetic approach

**Figure 1:**
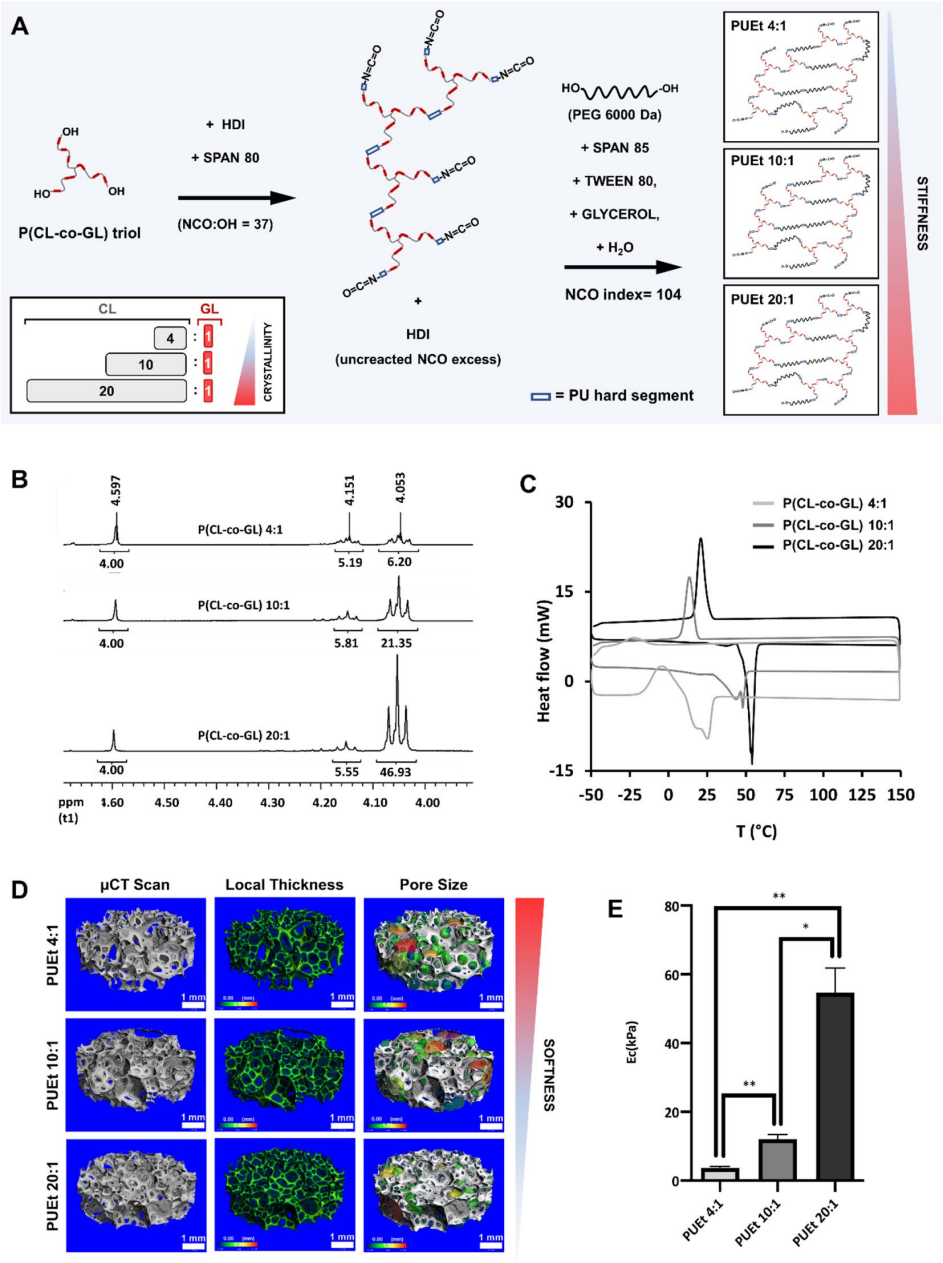
(**A**) Schematic representation of the synthetic route of 3 PUEt scaffolds of different stiffnesses, starting from 3 polyester triols: P(CL-co-GL) of different CL:GL ratios and accordingly of different crystallinities. Physicochemical properties of the 3 PUEt scaffolds formulations and the related precursors: (**B**) 1HNMR spectra of the 3 polyesters P(CL-co-GL) 4:1, P(CL-co-GL) 10:1, P(CL-co-GL) 20:1 showing correspondence between the ratio between CL and GL in the final products compared to the reactants; (**C**) DSC traces overlay of the 3 polyesters showing different thermal properties as a functions of the ratio CL: GL; (**D**) µCT scan micrograph (up), graphical rendering of the local thickness (middle) and of the pore size (bottom) of the 3 scaffolds formulations PUEt 4:1, PUEt 10:1 and PUEt 20:1; (**E**) graphical representation of the compression elastic moduli (Ec) of the 3 scaffolds formulations, showing statistically significant difference between: PUEt 10:1 and PUEt 20:1 (*p < 0.05), PUEt 4:1 and PUEt 10:1 (** = p < 0.025) and PUEt 4:1 and PUEt 20:1 (**p < 0.025).

Investigating the impact of the mechanical microenvironment on the in vivo performance of a scaffold implies the ability of fine tuning of the scaffold mechanical properties without changing or altering the remaining physicochemical characteristics which may affect the biological response. The mechanical properties of a crosslinked polyurethane foam can be modulated by varying the degree of crosslinking^19^, pore dimension^20^ or the ratio between hard and soft segments holding together the macromolecular structure^21^. Such strategies are unsuitable for the purpose of this study since they dramatically impact the scaffold internal morphology, density and water uptake capability^22^. The proposed approach focuses on the degree of crystallinity of the soft segments (*P(CL-co-GL)*) covalently bound to the *PUEt* network that exploit the ability of PCL segments to form semi-crystalline domains, as previously reported in May-Hernández et. al. 2011 and Rueda-Larraz et al.^23,24^. Briefly, 3 star-like polyester triols obtained by ring opening polymerization (ROP) of CL and GL (using glycerol as initiator) were synthesized with CL:GL ratios of 4:1; 10:1 and 20:1. By keeping the ratio between the initiator and the monomer constant, we were able to maintain the same average molecular weight of the 3 polyesters (Table 1). The obtained polyesters were end-capped by HDI using the same molar excess (i.e. eq. NCO/eq. OH = 37) and crosslinked and foamed using NCO index 104 (Fig. 1A)^25^. The physicochemical properties of the 3 scaffolds were assessed to verify the validity of the synthetic approach prior to the biological evaluation study in vivo. Structural analysis by ^1^HNMR spectroscopy of the 3 polyesters showed that the ratio CL:GL in the final product matched that in the feed (Fig. 1B, Fig. S1, Table 1, Table S1). The molecular weights of the 3 polyesters were confirmed to be similar by SEC (Table 1). The crystallinity, which was expected to modulate the stiffness of cross-linked PUEt-based scaffold, was assessed by DSC. P(CL-co-GL) 4:1 was completely amorphous while the polyesters: P(CL-co-GL) 10:1 and P(CL-co-GL) 20:1 exhibited 48% and 64% crystallinity, respectively (Fig. 1C).

**Table 1:** P(CL-co-GL) triols data table. [GL/CL]_HNMR_ i.e. the molar ratio between GL and CL for each polymer, was semi-quantitatively calculated from 1HNMR spectra of each polyester triol by integrating the areas under the peaks: 4.6 – 4.74 ppm (–O–C**H**_**2**_–COO–) of GL and 4.18 ppm (–(CH_2_)_4_–C**H**_**2**_–OOC–CH_2_–COO–) of CL (supplementary data: Fig. S1 and Table S1). The average number molecular weight was calculated by SEC analysis, using PS standard kit for system calibration. PD index is calculated as the ratio between the average number m.wt (M_n_) and the average weight m.wt (M_w_).

Polyester Name	*P(CL-co-GL) 4:1*		*P(CL-co-GL) 10:1*		*P(CL-co-GL) 20:1*	
Monomers*	*CL*	*GL*	*CL*	*GL*	*CL*	*GL*
Weight [g]	158.4	39.6	180	18	188.57	9.43
mol	1.388	0.341	1.564	0.154	1.651	0.081
w/w %	78.87%	19.72%	89.62%	8.96%	93.89%	4.69%
[GL/CL]_Feed_	0.25		0.10		0.05	
[GL/CL]_HNMR_	0.19		0.11		0.05	
Mn (SEC, PS standard)	7450		7400		7000	
PD index	1.45		1.49		1.53	

*CL* = ε-caprolactone, *GL* = Glycolide.

Compression tests on the 3 PUEt-based scaffold formulations, obtained by crosslinking said NCO-end capped polyesters, showed significant increase in the compression elastic modulus by increasing the amount of CL in the polyester (Fig. 1E, Table 2). By changing the ratio between amorphous and crystalline domains of the polyesters, we were able to successfully obtain 3 crosslinked PUEt-based scaffold formulations with different stiffness but of similar pore dimension and density (Fig. 1D, Table 2). All the scaffolds tested in this study were treated with poly(L-lysine) (PLL) to promote cell adhesion on the matrices surface and to minimize possible differences between the test scaffolds in terms of non-specific cell-biomaterial interaction^26^.

**Table 2:** A correlation between the polyester triol used in the synthesis of each PUEt scaffold formulation and the compression elastic modulus (Ec), porosity and density.

Polyester Name	PUEt scaffold formulation	PUEt -Average Ec (kpa)	PUEt -Porosity* %	PUEt -Density** (kg/m3)
P(CL-co-GL) 4:1	PUEt 4:1	3.6 (± 1.0)	95.80	38.8 (± 1.1)
P(CL-co-GL) 10:1	PUEt 10:1	12.0 (± 3.3)	96.02	39.1 (± 1.9)
P(CL-co-GL) 20:1	PUEt 20:1	54.7 (± 16.0)	95.52	37.9 (± 1.3)

* Measured by means of µCT scan.

** ASTM D3574 – 17: Standard Test Methods for Flexible Cellular Materials—Slab, Bonded, and Molded Urethane Foams.

#### In vivo tests

##### Macroscopic examination

No abnormalities were detected in terms of animal weight and food consumption during a 3-month observation period. After sacrifice, all scaffolds were retrieved from the implantation site except for 1 animal from the group: *PUEt-P(CL-co-GL) 10:1*.

##### Histological examination

**Figure 2:**
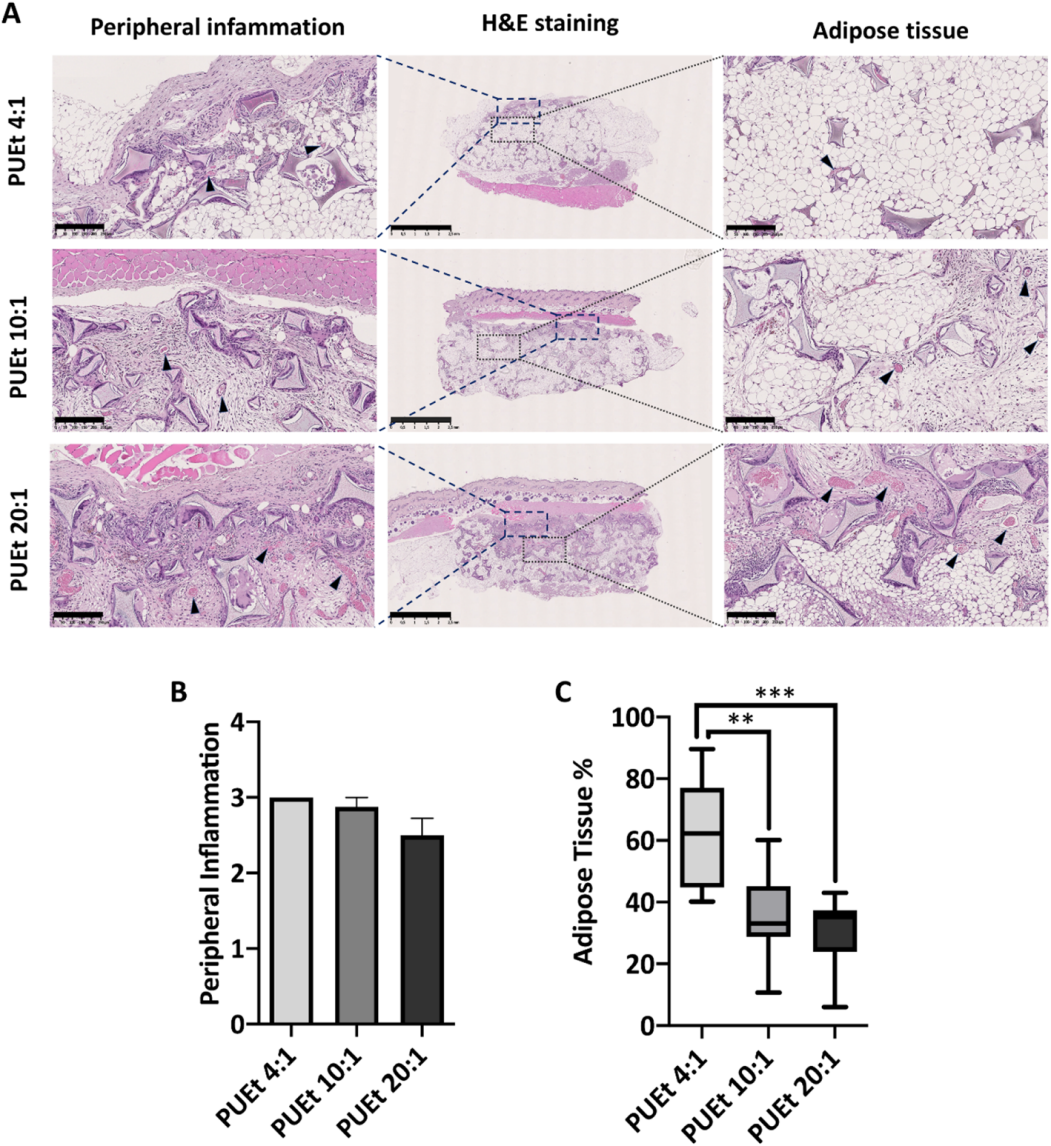
(**A**) representative H&E stained histological images of PUEt 4:1, PUEt 10:1, and PUEt 20:1. In the middle: low-magnitude images showing the entire explanted scaffolds after 3 months of subcutaneous implantation. Subregions were selected to show peripheral inflammation and adipose tissue (dashed blue and dotted gray, respectively) (scale bar, 2.5 mm). Left and right columns display higher magnification images representing the peripheral inflammation and adipose tissue in each group, respectively. Vascular tissue is indicated by black arrowheads, scale bar = 250 µm. (**B**) graphical representation of peripheral inflammation of the 3 groups: PUEt 4:1, PUEt 10:1, and PUEt 20:1 (scoring system described in paragraph 4.12, Table 4 and Table S2). No significant difference between the three groups was found, in terms of peripheral inflammation. (**C**) Graphical representation of the adipose tissue % for the 3 groups: PUEt 4:1, PUEt 10:1, and PUEt 20:1. The semi-quantification of adipose tissue % was carried out according to the method described in paragraph 4.14 and in Fig. 3. Statistically relevant differences in terms of % of adipose tissue was found between PUEt 4:1 vs PUEt 10:1 (**p < 0.025) and PUEt 4:1 vs PUEt 20:1 (***p < 0.01).

**Figure 3:**
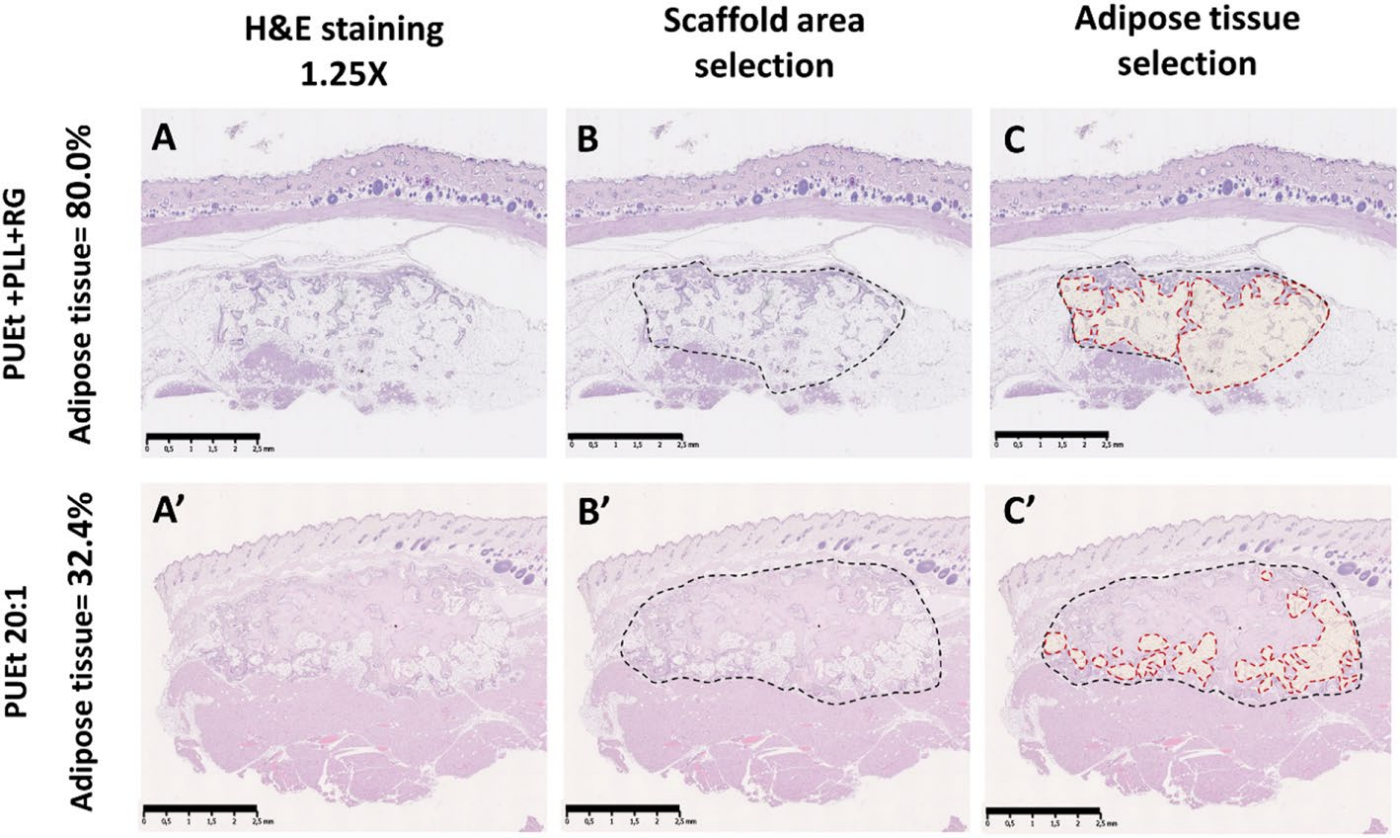
Schematic representation of the method adopted for semi-quantification of adipose tissue (%). Scanned histological sections was digitally processed by means of NDP.view2 software (Hamamatsu Photonics K.K., Hamamatsu City, Japan) to obtain (1.25 × magnitude) histological images. (**A**,**A**’) H&E stained histological images belonging to the replicas from the experimental groups: PUEt + PLL + RG (an example for high-adipose tissue %), PUEt 20:1 (an example for low-adipose tissue %), respectively; (**B**,**B**’) manual selection of the scaffolds region into the histological images, circumscribed by the black dashed line, to calculate the pixels corresponding to the scaffolds area (region of interest ROI) by means of analysis program ImageJ v1.52a (National Institutes of Health, Bethesda, Maryland, USA). The scaffold/host tissue interface is easily detectable where the polymeric matrix is no longer present. (**C**,**C**’) The regions of adipose tissue inside the selected scaffold regions, circumscribed by the red dashed line, were selected exploiting the characteristic shape of adipose cells (rounded white) which allowed to distinguish adipose tissue from the remaining types of soft tissue colonizing the scaffold. The sum of pixels from adipose tissue regions within the same ROI is multiplied by 100 and divided by the pixels of the ROI to calculate the % of adipose tissue in each histologic section. Scale bars = 2.5 mm.

Capsule formation around the scaffold was observed to be partial and discontinuous in almost all groups in the vicinity of muscular tissue (either the superficial subcutaneous muscle or the deep dorsal muscles). No capsule formation was observed on the lateral sides of the scaffold. No statistically significant differences among the 3 groups were observed in terms of capsule formation (Fig. 2A). The foreign body response was characterized by moderate amounts of macrophages and multinucleated giant cells surrounding the walls of the scaffold pores (indicated as peripheral Inflammation Fig. 2A, left column) in association with chronic inflammatory infiltrates represented by macrophages, lymphocytes, plasma cells, PMN in all the 3 groups without significant differences (Fig. 2B). In the central areas of the scaffold the colonizing tissue was associated with moderate presence of inflammatory cells, lymphocytes and macrophages in all the 3 groups without differences. Minimal apparent infiltration into the interior of the scaffolds was generally observed in all the experimental groups. No significant differences between all the 3 groups were observed in terms of presence of microhemorrhages. The % of adipose tissue to the total connective tissue infiltrating the scaffolds was semi-quantified by means of digital elaboration of the HE stained histological sections, according to the method described in paragraph 4.14 and in Fig. 3. Histological staining by Oil Red O was not possible due to the high affinity of the scaffold polymeric matrix for the dye. Nevertheless, thanks to the characteristic morphology of adipose tissue (rounded white), we were able to identify the adipose tissue infiltrates throughout the scaffold in the HE-stained histological sections. All the experimental groups showed similar performance in terms of foreign body response and microhemorrhages (Fig. 2A,B) but exhibited an increasing trend in the % adipose tissue as the scaffold stiffness decreased (Fig. 2C). The highest adipose tissue % was observed in *PUEt-P(CL-co-GL) 4:1,* where the elastic modulus was the lowest among the tested groups (Ec = 3.5 (± 1.0) kPa).

### Part II: The effect of biochemical manipulation by means of local release of a PPAR-γ agonist molecule on adipose tissue formation

#### RG loading to PUEt 10:1 scaffold and local release.

The *PUEt 10:1* scaffold was selected for this experimental tranche as it represents a midpoint among the 3 formulations in terms of scaffold softness (Table 2). Thus, the impact of biochemical manipulation by local release of PPAR-γ agonist molecules was elucidated in a neutral biomechanical conditioning scenario.

**Figure 4:**
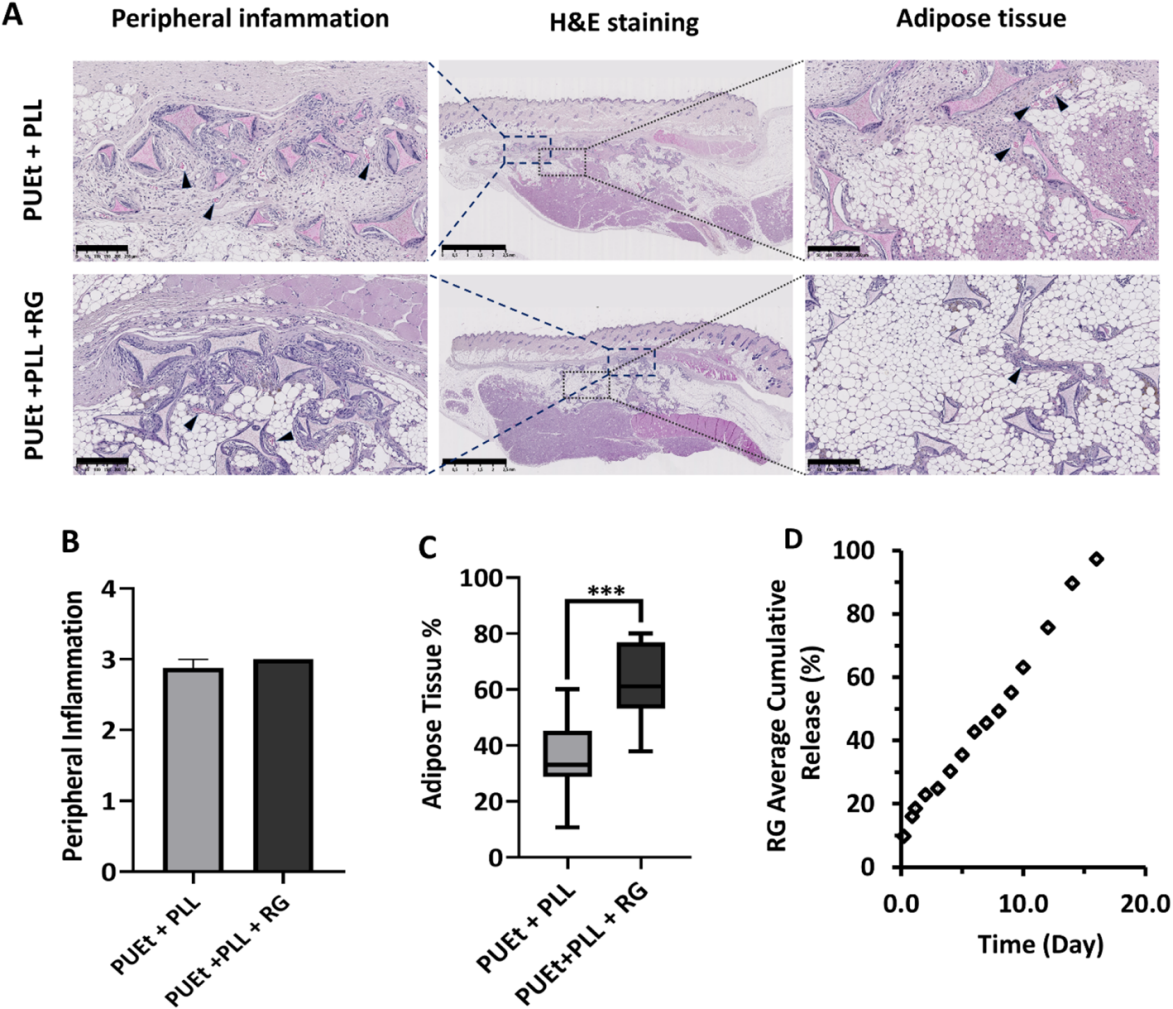
(**A**) representative H&E stained histological images of PUEt + PLL (control) and PUEt + PLL + RG. In the middle: low-magnitude images showing the entire explanted scaffolds after 3 months of subcutaneous implantation. Rectangular regions were selected to show peripheral inflammation and adipose tissue (dashed blue and dotted gray, respectively), scale bar = 2.5 mm. Left and right columns are high-magnitude selected images representing the peripheral inflammation and adipose tissue in each group, respectively. Vascular tissue is indicated by black arrowheads (scale bar, 250 µm). (**B**) graphical representation of peripheral inflammation of PUEt + PLL (control) and PUEt + PLL + RG. No significant difference between the 2 groups was found, in terms of peripheral inflammation. (**C**) Graphical representation of the adipose tissue %. The semi-quantification of adipose tissue % was carried out according to the method described in paragraph 4.14 and in Fig. 3. Statistically relevant differences in terms of % of adipose tissue was found between PUEt + PLL + RG and PUEt + PLL (control) (***p < 0.01). (**D**) Graphical representation of the in vitro cumulative release of RG from PUEt + PLL + RG, under physiological conditions. The release profile is characterized by a 9.35% initial bust effect and 5.50% daily release. The in vitro release proceeded for 16 days until complete release of RG from the scaffold.

The amount of RG loaded to the scaffolds as well as the amount of release medium in vitro were chosen to resemble the RG concentration in Han Wistar female rats on the bases of a systemic release and considering the total blood volume of the animal model. In vitro, the release profile of RG was characterized by an initial burst effect of 9.35%, a common phenomenon related to high specific surface area of the scaffold which facilitates the diffusion of the loaded molecules located in the outer substrates of the matrix to release medium. RG exhibited constant daily release of 5.5%/day and was completely released from the matrix after 16 days (Fig. 4D). Considering that the average body weight of a Han Wister female rat was 220–260 g, the calculated total blood volume (TBV) was 14 to 16 mL^27^. Based on the in vitro release profile, the simulated initial burst of RG in vivo corresponds to 0.84–1 µM RG (equivalent to 0.019–0.022 mg/kg) and the simulated daily release to corresponds 0.5–0.6 µM RG (equivalent to 0.011–0.013 mg/kg/day). This concentration appears far below the therapeutic dose for the systemic activation of peroxisome proliferator–activated receptor γ (PPAR-γ) commonly used for insulin sensitization for diabetes treatment (i.e. 3–10 mg/kg/day in rats)^28^. However, in the local environment, the initial burst corresponds to 22–44 µM and the daily release corresponds to 13- 26 µM. These concentrations should be sufficient to activate PPAR-γ receptors on the cells invading the scaffold and in the adjacent surrounding tissue^29,30^.

#### In vivo test

##### Macroscopic examination

No abnormalities were detected in terms of animal weight and food consumption during a 3-months observation period.

##### Histological examination

No significant differences were found between groups for the following parameters: capsule formation, inflammation in the scaffold interior and microhemorrhages. The foreign body response associated with macrophages, lymphocytes, plasma cells, and PMN cells was moderate in the group PUEt + PLL. Notably, no significant difference was observed between the two groups in terms of peripheral inflammation L/P/M/G (Fig. 4A,B). The PUEt + PLL + RG and PUEt + PLL (control) groups had a high mean colonization (%) of scaffolds (> 85%). Colonization > 90% was observed in 100% of the explants in group PUEt + PLL. A reduced amount of dense fibrous connective tissue was observed in the group PUEt + PLL + RG compared to PUEt + PLL (control) (Fig. 4A). The amount of adipose tissue was semi-quantified as described in paragraph 4.14 and in Fig. 3. The average adipose tissue % was significantly higher in PUEt + PLL + RG scaffolds compared to PUEt + PLL ones (Fig. 4A,C). Since the two groups share the same PUEt formulation, mechanical and morphological properties, we believe that the significant increase in the % of adipose tissue in PUEt + PLL + RG scaffolds is due to the local release of RG to the tissue colonizing the scaffold via specific activation of PPAR-γ receptors.

## Discussion

In this study we investigated the importance of biomechanical and biochemical cues for conditioning the 3D regenerative microenvironment towards soft tissue formation through two separate set of experiments in vivo. In the first part of this study, a family of 3D microenvironments with different stiffnesses was created using a 3D PUEt scaffolds. In brief, 3 crosslinked porous PUEt scaffolds formulations were prepared with different stiffnesses but similar physicochemical properties (i.e., pore dimension, local thickness, density, specific surface area, ratio between soft and hard segments in the polymeric matrix) and implanted in Han Wistar female rats in an interscapular site beneath the fat pad. Their biological performance was evaluated by histological examination and by digital analysis (by means of dedicated software) in order to semi-quantify the amount of adipose tissue in the newly formed tissue inside the scaffolds. Our results showed that infiltration of adipose tissue significantly increased with decreasing scaffold stiffness. Thus, targeting the scaffold mechanical properties to that of adipose tissue was beneficial and inhibited fibrous tissue formation. This result aligns with previous findings reported by Young, D. Adam, et al., that mechanical cues play a key role in adipogenic differentiation^14^.

Scaffold fabrication for the regeneration of large portions of connective tissue can be achieved using additive manufacturing techniques, including 3D printing and filament deposition of thermoplastic biodegradable polymers^31^. The most commonly used biomaterials are semi-crystalline linear polyesters, such as polycaprolactone (PCL), polylactide, polyglycolide and their block co-polymers. These polymers allow rapid solidification of the extruded filament at room temperature, load bearing capability and precise fabrication of the final construct. Paradoxically, the mechanical properties of this family of polymers, in particular their excessive rigidity, affect the biological performance in vivo, because they are unable to supply adequate mechanical cues to induce differentiation of infiltrating cells to adipose or soft tissue^32,33^. In the very specific case of breast reconstruction, the challenges against the use of very soft polymeric biomaterials in the fabrication of scaffolds for the regeneration of large portions of adipose tissue are basically related to suboptimal capability to withstand the applied mechanical stress due to the normal patients’ activity, including prolonged load bearing periods (due to patients’ sleeping positions, for example) and the suboptimal capability to gain the original shape after deformation^34–36^. Crosslinking of soft amorphous segments can resolve the shape recovery issues^37,38^ but requires significant effort to enable the achievement of an appropriate micro-architecture (such as interconnected pores, channels or voids) necessary for cellular infiltration of the scaffold and maintenance of cell viability^39^. In our previous study, we explored the potential of soft crosslinked polyurethane porous scaffolds for the regeneration of adipose tissue by evaluating the resilience and elasticity in response to the ratio of hydrophilic/hydrophobic segments^16^. In Vannozzi, et al., we showed that this library of soft scaffolds with compression elastic moduli ranging from 10 to 30 kPa maintained their elastic behavior even after 1500 compression cycles at 25% of deformation with minimal compression set^17^. The manufacturing of medical grade biodegradable polymers is a sophisticated process, even more in case of very soft porous crosslinked scaffolds, since it involves the use of highly degradable soft segments which are crosslinked and foamed simultaneously. The arrival of finely tuned crosslinked soft scaffolds to market, including soft biodegradable polyurethanes, requires important technical and economical effort to the address scalability issues and conformity to the quality standards for the manufacturing of medical devices^3^. However, from the authors' point of view, this type of effort may significantly bring the development of definitive solutions for breast reconstruction on the verge of a breakthrough.

The second tranche of this study aimed to compare the mechanical conditioning effect with the impact of the biochemical signaling on in vivo performance using the developed 3D PUEt scaffold. While the capability of PPAR-γ agonists molecules to trigger adipogenic differentiation was extensively demonstrated in vitro^40,41^, currently there are very few studies exploring the effect of PPAR-γ activation by the local release of pharmacological agents from 3D scaffolds- on the modulation of cell response and tissue composition^42,43^. Herein, we performed gradual local release of RG, an FDA-approved drug for the treatment of diabetes, with potent and selective PPAR-γ agonist activity (EC_50_ = 18 nM)^44,45^. This experiment was designed to avoid systemic reactivity by adjusting the amount of released RG to be far lower than the therapeutic threshold but suitable for PPAR-γ local activation and induction of adipogenesis. Despite the fact that in vitro RG was completely released from the scaffold after 2 weeks of incubation in vivo, the release profile can be slowed by the complexity of the in vivo context that involves deposition of cells and blood vessels near the scaffold pores, deposition of extracellular matrix on the polymer walls and differential liquid flow though the scaffold compared with constant sink of release medium^46^. Accordingly, we considered the release kinetics in vitro as a worst-case scenario to calculate the approximate maximum RG daily dose, systemically and locally, prior to implantation. Given that the scaffolds used in both of the tested groups in this experimental tranche were identical in physicochemical and morphological characteristics, the significant increase of adipose tissue % in the explanted RG-loaded implants is due to PPAR-γ activation, the triggering factor for adipogenesis^47^. In addition, results confirmed the mechanical and biochemical conditioning equivalency in promoting adipose tissue formation under the conditions tested, suggesting that adequate mechanical signaling would be sufficient to boost adipogenesis by drastically influencing cell differentiation.

## Materials and methods

### Materials

Glycerol (BioUltra, ≥ 99.5%, Sigma-Aldrich), glycolide (GL, ≥ 99%, ITV Denkendorf Gmbh), ε-caprolactone (CL, > 95%, Sigma-Aldrich), poly(ethylene glycol) average molecular weight 6000 (PEG6000, 95%, Sigma-Aldrich), hexamethylene diisocyanate (HDI, 98.85%, Vencorex), sorbitane monoleate (Span 80, 61.9%, Sigma-Aldrich), sorbitane trioleate (Span 85, 61%, Sigma-Aldrich), polysorbate 80 (Tween 80, CRODA), dibutyl tin dilaurate (DBT, 95%, Sigma-Aldrich), tin(II) 2-ethylhexanoate (92.5–100%, Sigma-Aldrich), rosiglitazione (RG, ≥ 98%, Sigma-Aldrich), methylene chloride (≥ 99.8%, Carlo Erba), and poly-L-lysine solution 0.01% (PLL 0.01%, mol.wt. 70–150 kDa, Sigma-Aldrich) were used without further purification unless otherwise indicated.

### Syntheses of P(CL-co-GL) triols

The 3 polyester triols were prepared by ring-opening polymerization of CL and GL, using glycerol as initiator, and tin(II) 2-ethylhexanoate as catalyst. The reaction scheme is shown in Fig. 1A.

Glycerol (26.4 g, 0.29 mol) was charged to a round-bottom two-necked flask equipped with magnetic stirrer and reflux condenser followed by predetermined amounts of GL and CL as listed in Table 1. After flushing with dry N_2_, the reactive mixture was refluxed at 60 °C using an oil bath until complete solubilization, at which time the system was dried under reduced pressure of 0.01 mbar while stirring at 500 rpm. After 12 h under vacuum, 1.58 mL of tin(II) 2-ethylhexanoate (ρ = 1.25 g/mL, 4.9 mmol) were added to the mixture. The reaction proceeded for 4 h at 150 °C under a dry N_2_ atmosphere. After completion, the reaction system was dried under vacuum at 120 °C for 12 h. The polymer was thus obtained as a viscous yellowish liquid (at 120 °C) and used without further purification.

^**1**^**H NMR** (CDCl_3_ as solvent, 400 MHz): δ = 1.2 – 1.9 (––CH_2_–C**H**_**2**_–CH_2_–); δ = 2.3 ppm (–OOC–C**H**_**2**_–(CH_2_)_4_–OOC–(CH_2_)_5_–); δ = 2.44 ppm (–OOC–C**H**_**2**_–(CH_2_)_4_–OOC–CH_2_–O–); δ = 3.65 ppm (–CH_2_–C**H**_**2**_–OH); δ = 3.75 ppm (–OOC–C**H**_**2**_–OH); δ = 4.07 ppm (–(CH_2_)_4_–C**H**_**2**_–OOC–(CH_2_)_5_–); δ = 4.18 ppm (–(CH_2_)_4_–C**H**_**2**_–OOC–CH_2_–COO–); δ = 4.6 – 4.74 ppm (–O–C**H**_**2**_–COO–);

**IR** (cm^-1^): 2943(ν_as_ (CH_2_), w); 2864(ν_s_ (CH_2_), w); 1722(ν_(C=O ester)_, s); 1240(ν_as (O-C–C)_,w); 1155(ν_as (C–O–C ester)_, s); 1045(ν_s (C–O–C ester)_, m); 733(δ_(C-H)_, w).

### Preparation of quasi-prepolymers

Each of the 3 polyester triols: P(CL-co-GL) 4:1; P(CL-co-GL) 10:1 and P(CL-co-GL) 20:1 was end-capped with NCO group according to the procedure described in EP3594253A1^48^ and graphically shown in Fig. 1A. All the 3 polyester triols were converted to NCO-terminated quasi-prepolymers under the same reaction conditions: temperature, polyester addition rate, reactors type, mixing speed and amount and using a molar excess NCO:OH of 37 in order to attribute any difference in the scaffold characteristic to the sole ratio between CL and GL. Briefly, a mixture of hexamethylene diisocyanate (1330.5 mL, 8.26 mol) and Span 80 (29.5 g, 0.069 mol) was introduced in a double-necked round-bottom flask, equipped with reflux condenser and magnetic stirrer. The mixture was then heated to 60 °C. The polyester triol (1.54 kg, 0.22 mol) was heated to a melt, added to a dropping funnel and dropped at the rate of 2 mL/min into the reaction flask while stirring the mixture vigorously at 500 rpm. The mixture was reacted for 1.5 h at 60 °C to complete conversion without further stirring. The quasi-prepolymers were employed in the next crosslinking reaction as it is, without further purification.

### Synthesis of crosslinked PUEt-based scaffolds

The 3 PUEt scaffold formulations were synthesized by crosslinking 3 quasi-prepolymers under the same conditions. The nomenclature system for the 3 polyurethane-ester-ether (PUEt)-based scaffold formulations tracks the type of polyester type and the ratio between CL and GL as described in Fig. 1A and Table 2. For example, the acronym PUEt 4:1 indicates a Poly(Urethane-Ester-ether) obtained from crosslinking the polyester P(CL-co-GL) where the ratio between CL and GL is 4:1.

A polyol solution (Mix A) consisting of glycerol, water, span 85, tween 80, PEG 6 K was prepared according to the fractions shown in Table 3. The polyol solution was then heated in an oven at 100 °C until homogeneously melted. In the meanwhile, the quasi-prepolymer solution (Mix B) was kept in oven at 60 °C in a covered polypropylene tray. 28 g of Mix A was introduced in a polypropylene beaker containing 100 g of Mix B, and mechanically stirred until homogeneous, before adding 0.03 mL of DBT (the catalyst). Subsequently, the reactive blend was mixed mechanically for 30 s at 1000 rpm. After removing the mechanical stirrer, foam expansion proceeded for 20 s, reaching the final gel-point due to crosslinking. The density of the 3 raw foams was in the range 35–40 kg/m^3^. The volume of the raw foam blocks obtained using the reported amount of reactants was 4L.

**Table 3:** The PUEt formulations data. All the 3 formulations: PUEt 4:1; PUEt 10:1; PUEt 20:1, were synthetized according to the same experimental condition except for the CL:GL ratio in each polyester, as described in Table 2 and Fig. 1A.

Formulation data	Value
Isocyanate index*	106
PEG 6 K (pphp**)	21,16
Poly(e-CL-co-Gl) triol (pphp)	64,24
Glycerol (pphp)	7,6
Water (pphp)	3,34
Span 80, Sorbital monooleate (pphp)	1,23
Span 85, Sorbital trioleate (pphp)	0,24
Tween 80 (pphp)	2,18
DBT (pphp)	0,11
Density of free-rise foam (kg/m3)	35–40

*((eq NCO)/(eq OH)∙100); ** = pphp, parts per hundred polyol.

After solidification, the raw foams were kept in an oven at 40 °C for 24 h to complete the reaction conversion. Cylindrical samples (⌀ = 10 mm, h = 4 mm) were then cut from the raw foam blocks by means of metallic puncture and purified in methylene chloride using a Soxhlet extractor^49,50^. The extraction process proceeded for three days. Methylene chloride was removed from the purified samples by evaporation under reduced pressure (0.001 mbar) for five days in vacuum oven at 40 °C. The dried samples were finally stored in a dry and dark place at 4 °C.

### Surface functionalization of PUEt scaffold matrices with Poly(L-lysine)

Purified PUEt cylindrical samples underwent plasma-oxygen treatment (40 W, 80 s) in a plasma reactor (Colibrì, Gambetti Kenologia), using O_2_ as ionizing gas. Subsequently, 314μL of poly(L-Lysine) solution (0.01% w/w, MilliQ water) were added dropwise over each sample. The solution was allowed to bind to the matrix surface for 30 min. The unbound part was removed by washing the functionalized PUEt-based scaffolds with milliQ water, which in turn was removed by freeze-drying for 24 h.

### RG loading to PUEt 10:1 scaffold and in vitro release kinetics

A stock solution was prepared by dissolving Rosiglitazone (RG) in methylene chloride at a concentration of 0.99 mg/mL. Six cylindrical samples with diameter = 1 cm, height = 0.4 cm, volume = 0.31 cc (i.e., the same dimensions as those employed in the in vivo test) were used for the drug loading experiment. For each sample, 53 μL of the stock solution was added dropwise in order to load 52.8 μg of RG. Subsequently, the solvent was removed from the scaffold matrix by evaporation under reduced pressure (0.001 mbar) for 72 h in a vacuum oven at 40 °C. RG- loaded scaffolds were then incubated in 15-mL Falcon vials filled with 10 mL PBS 1X and incubated at 37 °C. At each time point (namely: 2, 3, 4, 5, 7, 9, 72 h; and 4, 5, 6, 7, 10, 11, 12, 13, 14 days), 0.1 mL of the release medium was withdrawn for analysis by UV spectroscopy to determine the concentration of the released RG overtime. The volume of the release medium was kept constant during the whole incubation period. The Lambert–Beer constant was experimentally determined by plotting the UV maxima in absorbance (λ_max_ = 313 nm) at 10 different concentrations. The UV–Vis spectra were acquired in the 390–800 nm range using a Cary 100 Spectrophotometer (Agilent). All the measurements were run at room temperature (25 °C) and were acquired using an ultra-low volume quartz cuvette with a 4-mm path length and a 1 × 2-mm window. 6 PLL-coated PUEt 10:1 scaffolds with the same geometry and dimension were used as control samples (blank system) during UV measurements.

### Fourier-transformation infrared (FTIR) spectroscopy

Structural elucidation of the chemical composition of PUEt scaffold matrices was carried out using a Cary 660 FTIR spectrometer from Agilent Technologies equipped with a Germanium crystal ATR accessory. The FTIR spectra of the sample were taken in the MIR range (400–4000 cm^-1^) with a resolution of 2 cm^-1^.

### Size exclusion chromatography (SEC)

The average number molecular weights (Mn) and the polydispersity indices (PD = Mn/Mw) of the 3 polyester triols was determined by SEC analyses, using a chromatograph composed of a Knauer isocratic pump and a mobile phase consisting of THF with a flow of 1.0 mL/min. The stationary phase consisted of two columns in series maintained at 35 °C: Resipor-PL (400,000–500 Da) and Tosoh (20,000–500 Da). The employment of a multidetector: Light Scattering (at 7° and 90°), refractive index and a Viscotek differential viscometer, combining signals processed with Omnisec v4.6 software (Malvern Panalytical Ltd, UK), allowed data correlation between the molecular weights (Mn, Mw, Mz) and the absolute intrinsic viscosity. Polystyrene standards were used to calibrate the system.

### DSC test and calculation of crystallinity %

The analyses were carried out using a differential scanning calorimeter previously calibrated with pure indium standard (Q2000-TA Instruments, USA). Data analysis were elaborated by Platinum TM software (TA Instruments, USA). 10-mg samples were placed in 40-mL Al crucible then closed hermetically by Al lid prior to placement in the calorimeter. A first heating cycle was carried out at the rate of 10 °C/min from − 70 °C to 250 °C to erase thermal history of the polymers and to eliminate traces of water from the sample. The cooling cycle proceeded at the rate of 10 °C/min, from 250 °C to -70 °C and the second heating cycle was carried out at the same conditions of the first one.

The crystallinity (%), of the 3 polyester triols: P(CL-co-GL) 4:1; P(CL-co-GL) 10:1 and P(CL-co-GL) 20:1, was calculated as in Eq. 1:

1 $${\text{Crystallinity}}\;\left( \% \right) = \left( {\Delta {\text{H}}_{{\text{m}}} {-}\Delta {\text{H}}_{{\text{c}}} } \right)/\Delta {\text{H}}_{{{\text{m}}0}}$$

where ΔH_m_ is the heat of melting J/g of the crystalline domains in the test semi-crystalline polymer occurring during the heating cycle of the DSC test, ΔH_c_ is the heat of crystallization J/g occurring during the cooling cycle, and ΔHm0 is the heat melting enthalpy of a fully crystalline PCL of similar molecular weight to the test polymers. ΔHm0 was 135 J/g as reported by Khambatta^51^ and applied by Nagata^52^. Since no crystallization due to cooling was detected in all the tested polymers, the crystallinity % was calculated by dividing ΔH_m_ by ΔH_m0_.

### Compression tests

Mechanical properties under compression were measured using a testing machine (model BR EMT503 A, MP Strumenti, Pioltello (MI), Italy) equipped with a 100 N load cell (transcell, model BAB-10 M) and operated at a crosshead speed of 1.3 mm/min. Cylindrical samples of 10 mm diameter and 10 mm height were analyzed according to the ISO 604:2002(E) standard. The compressive modulus (Ec) was calculated from the slope of the stress–strain curve in the elastic region (i.e., 0 to 5% strain). The yield point was determined from the stress/strain curve as the flection point between the elastic and plastic deformation. The compressive stress was calculated at 90% strain. Measurements were carried out at room temperature (37 °C) using cylindrical samples incubated for 1 h in milliQ water.

### Morphological characterization of PUEt scaffolds by µCT scan

The test was carried out using a commercially available cabinet cone-beam µCT (µCT 50, SCANCO Medical AG, Brüttisellen, Switzerland) originating from a 4 lm focal-spot X-ray tube. The photons are detected by a CCD- based area detector and the projection data are computer-reconstructed into a 3072X3072 image matrix. The scan image was segmented with a first threshold for polymeric material with x-ray absorption > 0.2 [1/cm]. Objects were superimposed, and 3D rendering were produced. The density distribution histogram of the scaffolds was obtained by binning all its voxels by density (excluding zeros). All image processing was carried out using IPL (Scanco Medical AG).

### In vitro evaluation of the cytocompatibility test

The cytotoxicity of the PUEt scaffolds was assessed through indirect cytotoxicity testing, using 3T3-L1 murine cells (ECACC No 86052701) as in vitro model, and following the standard practice UNI EN ISO 10,993–5. Briefly, sample material was obtained from the purified scaffolds by cutting a disk (d = 6 mm, h = 2 mm), which was subsequently incubated for 24 h at 37 °C, 120 rpm in cell culture medium (Dulbecco’s Modified Eagle Medium, 10% v/v bovine calf serum, 100ug/ml Penicillin–Streptomycin, and 2 mM L-glutamine). 3T3-L1 cells were seeded in 96-multiwell (7,6 × 103 cells/well) and cultured until 70% confluent in 150 µL of culture medium. Then, culture medium was replaced with medium eluates extracted from the PUEt samples as described above, with additional triplicate control wells with non-eluate cell culture medium as positive control, and DMSO as negative control. Cells were then cultured for further 24 h. Cell viability was monitored for 72 h using an inverted optical microscope (Zeiss, Primovert. Carl Zeiss Microscopy GmbH). A scoring system based was used to assess cytotoxic effects on the incubated cells on observation, as described in Table 4. Scaffolds were employed in the in vivo test if and only if the % of live cells incubated with the extracted eluates was from 95 to 100%.

**Table 4:** Scoring system for the evaluation of the cytocompatibility test in vitro.

Scoring	Live Cells %
3	95 to 100
2	50 to 95
1	5 to 50
0	0 to 5

### In vivo tests

The study reported in this manuscript, protocols and experimental designs on animals were reviewed and approved by the Animal Welfare Body (Organismo Preposto al Benessere degli Animali OPBA) and authorized by the National Competent Authority (Italian Ministry of Health, 200–2015-PR, March 31^st^ 2015).

All animal procedures and methods were carried out in accordance with the current Italian regulations (Legislative Decree March 4^th^ 2014, n. 26, enforcing the EU Directive 2010/63 of the European Parliament and the Council of September 22^nd^ 2010 on the protection of animals used for scientific purposes).

The study reported in this manuscript was carried out in compliance with the ARRIVE guidelines (Animal Research: Reporting of In Vivo Experiments).

Female Han Wistar rats (Charles River Laboratories) aged 14 weeks (weight 220–260 g) were used for this study. To avoid or minimize pain and distress, about 30 min prior to surgery tramadol 4 mg/kg was administered by subcutaneous injection to provide a therapeutic level of systemic analgesia. While under anesthesia (an aqueous mixture of ketamine, xylazine and cepromazine, intramuscularly administered), the rats were surgically prepared for subcutaneous implantation. The graft was positioned into the adipose tissue, reaching the interscapular region after skin incision and blunt dissection. The subcutaneous implantation (particularly near the adipose tissue: i.e., interscapular area) has been selected as it is comparable to the one envisaged in humans. Scaffold’s geometry was cylindrical having ~ 1 cm diameter, ~ 0.4 cm height and ~ 0.31 cc volume. The experimental design is summarized in Table 5.

**Table 5:** The experimental design of the in vivo tests.

Experiment Package	Test Group	Description			No. of Animals
		Polyester Triol Type	Scaffolds Average Compression Elastic Modulus (kPa)	Surface functionalization; PPAR-δ agonist loading	
1- The impact of the scaffold mechanical on the scaffold biological performance in vivo	PUEt 4:1	P(CL-co-GL) 4:1	3.5	PLL	6
	PUEt 10:1	P(CL-co-GL) 10:1	12.0		8
	PUEt 20:1	P(CL-co-GL) 20:1	54.7		6
2- The impact of local release of PPAR-γ agonists on the scaffold biological performance in vivo	PUEt + PLL*	P(CL-co-GL) 10:1	12.0	PLL	8
	PUEt + PLL + RG		12.0	PLL; RG	6 (-1)

*The test group: PUEt 10:1 + PLL, belonging to the first experimental package was used as control group for the second experimental package.

After sacrifice, scaffolds together with the surrounding tissues were excised and fixed in 10% neutral buffered formalin. Formalin-fixed samples were embedded in paraffin wax, sectioned at 4 µm thickness, routinely stained with hematoxylin and erythrosine, then evaluated under a light microscope for the histological assessment of host reaction (fibrous encapsulation, extent and type of inflammatory cells, necrosis, nature and extent of tissue ingrowth into the pores, cell adhesion to the material, and cell infiltration into the material) according to the ISO 10,993–6 standard for biological evaluation of medical devices (Supplementary data Table S2).

### Adipose tissue semi-quantification

The % area of adipose tissue was quantified using the analysis program ImageJ v1.52a (National Institutes of Health, Bethesda, Maryland) Briefly, from 1.25X histological scanned images of the samples (obtained with NDP.view2, Hamamatsu Photonics K.K., Hamamatsu City, Japan) it was first selected the entire area occupied by the scaffold (Scaffold area, in pixels or mm^2^). All the areas identified with adipose tissue were then selected and summed, to obtain the total adipose area (Σ Adipo area) in pixels or mm^2^, as graphically illustrated in Fig. 3. The % area of adipose tissue was then calculated according to Eq. 2.

2 $$\% \;{\text{Adipose}}\;{\text{Tissue}}\;{\text{Area}} = \left( {\Sigma {\text{Adipo}}\;{\text{area}}} \right)/\left( {{\text{Scaffold}}\;{\text{area}}} \right) \cdot {\text{1}}00$$

### Statistical analyses

Data was analyzed using GraphPad Prism v8.3.1 (GraphPad Software Inc., San Diego, CA, USA). Histology inflammation results were presented as mean ± standard error (SEM) while data analysis was performed using Kruskal–Wallis test followed by Dunn’s post-hoc test for the evaluation of statistically significant differences. Compression test results were presented as mean ± standard error (SEM) while data analysis was performed by Brown-Forsythe and Welch test followed by Tamahane T2 post-hoc test to evaluate statistically significant differences among samples.

Results of % Adipose Tissue Area were plotted as box plots for better showing and comparing data distributions. Each box encompassed 25–75 percentiles, extending-lines covered all the values from minimum to maximum value, while the thick line was the mean of the values. One-way ANOVA together to Tukey’s post-hoc tests were used to evaluate statistical differences in Adipose Tissue % among the test groups in the first experimental tranche of the study, while unpaired Student’s t-test was chosen for the groups in the second one. The significance thresholds for all tests were set at 5% (*p < 0.05), 2.5% (**p < 0.025), and 1% (***p < 0.01).

## Conclusions

One of the major challenges of biomaterials in regenerative medicine is establishing the correct balance between in vivo performances, clinical translatability, and regulatory complexity. Adapting commercially available polymers appears attractive from a go-to-market perspective. However, customizing these polymers to a specific clinical application, such as adipose tissue regeneration, requires enormous efforts. On the other hand, ab initio material design informed by specific biological requirements and focusing on clinical translatability could be the most cost-effective strategy. To this aim, we investigated the role of biomechanical cues in the 3D regenerative microenvironment, by creating a gradient of 3D synthetic matrices with different stiffnesses using a PUEt platform. The advantage of this approach consists in performing fine tuning of the mechanical properties while keeping unvaried the remaining physicochemical and morphological characteristics. Our results show that: (1) reducing the scaffolds elastic modulus increases the proportion of adipose tissue in the scaffold and (ii) the effect of this mechanical conditioning was similar to that observed with biochemical manipulation by loading PUEt scaffolds with a PPAR-γ agonist molecule, in the conditions tested. These results suggest that adequate mechanical signaling could be sufficient to boost adipogenesis in vivo by influencing cell differentiation. Moreover, we hypothesize that mechanical conditioning, thanks to its persistence in the microenvironment, could be advantageous for the regeneration of clinically relevant tissue volumes over the release of biochemical cues, which is usually limited in time. This factor would be even more significant in large scaffolds where the regeneration process could take several months to be completed requiring continuous stimulation to the ingrowing tissue^53^. Overall, the authors believe that this work unveils a new avenue in the design of synthetic 3D ECMs for microenvironment conditioning, for the regeneration of large soft tissue able to compensate for the volume and function of resected tissues with practical and direct implications in reconstructive and cosmetic surgery.

## Supplementary Information

Supplementary Information.
