# Supplementary material for: Conditioning the microenvironment for soft tissue regeneration in a cell free scaffold
Source: Sci Rep. 2021 Jun 25;11:13310. doi: 10.1038/s41598-021-92732-9 (PMC8233309; doi:10.1038/s41598-021-92732-9)
Supplement: Supplementary file 1 — Supplementary Information. [file 41598_2021_92732_MOESM1_ESM.pdf]

Supplementary data

Title: Conditioning the microenvironment for targeted *in vivo* adipogenesis and soft tissue regeneration in a cell-free poly(urethane-ester)-based scaffold

Authors: Irini Gerges[*§1], Margherita Tamplenizza[*1], Federico Martello[1], Stefano Koman[1], Giulia Chincarini[1], Camilla Recordati[2], Mariacaterina Tamplenizza[1], Scott Guelcher[3], Maurizio Crestani[4], Alessandro Tocchio[§1].

* IG and MT contributed equally to this manuscript
§ Corresponding authors: Irini Gerges, E-mail: irini.gerges@tensivemed.com, Alessandro Tocchio, E-mail: alessandro.tocchio@tensivemed.com

Affiliations:

1 Tensive SRL. Via Timavo 34, Milan, Italy;
2 Dipartimento di Medicina Veterinaria. Università degli Studi di Milano, Via dell'Università, 6, 26900 Lodi, Italy;
3 Department of Chemical and Biomolecular Engineering, Vanderbilt University, PMB 351604 2301 Vanderbilt Place, Nashville, TN 37235-1604, USA;
4 Dipartimento di Scienze Farmacologiche e Biomolecolari – DiSFeB. Università degli Studi di Milano, Via Balzaretti, 9, 20133 Milan, Italy;

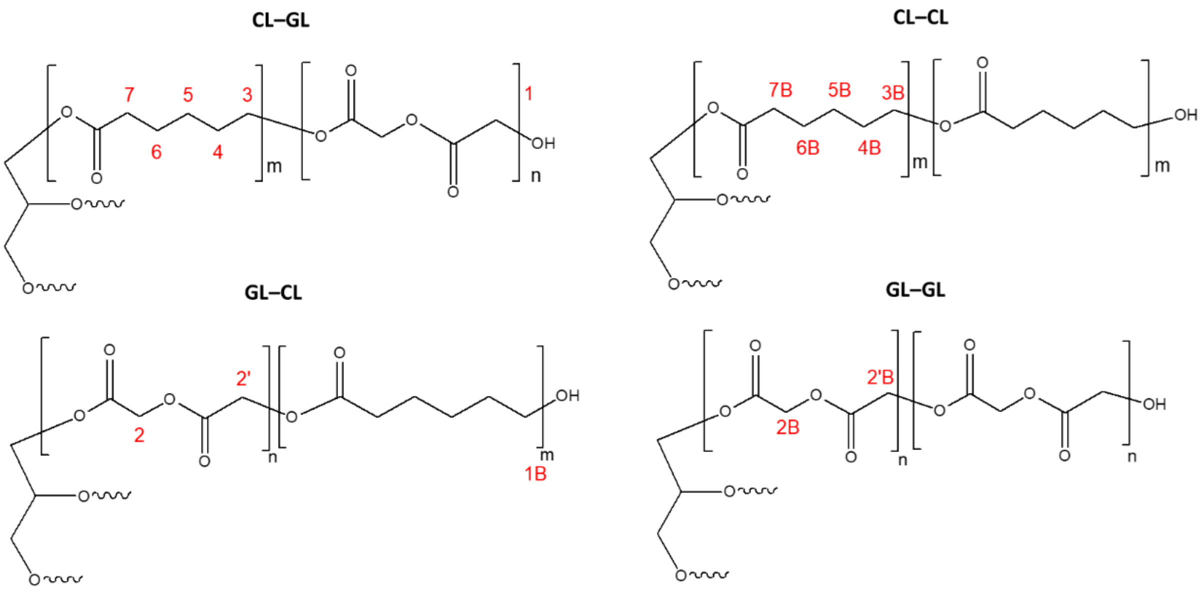

Figure S1. Chemical structure of P(CL-co-GL)triol. Only one branch of the star polymer is represented. The 4 alternations between CL and GL units: CL-GL; CL-CL; GL-CL; GL-GL were reported, where the relevant protons where numbered in red for structural elucidation by HNMR spectroscopy

<table>
<tr><th></th><th></th><th></th><th colspan="2">P(CL-co-GL) 4:1</th><th colspan="2">P(CL-co-GL) 10:1</th><th colspan="2">P(CL-co-GL) 20:1</th></tr>
<tr><th>Signals (ppm)</th><th>Assignment</th><th>Total H number</th><th>integral</th><th>norm. area</th><th>integral</th><th>norm. area</th><th>integral</th><th>norm. area</th></tr>
<tr><td>1.2- 1.9</td><td>4,5,6,4B,5B,6B (CL)</td><td>6</td><td>610.69</td><td>101.78</td><td>448.53</td><td>74.76</td><td>393.26</td><td>65.54</td></tr>
<tr><td>2.3</td><td>7B (CL)</td><td>2</td><td>100.22</td><td>50.11</td><td>100</td><td>50</td><td>100.24</td><td>50.12</td></tr>
<tr><td>2.44</td><td>7 (CL)</td><td>2</td><td>84.06</td><td>42.03</td><td>26.82</td><td>13.41</td><td>12.35</td><td>6.18</td></tr>
<tr><td>3.65</td><td>1B (chain end groups)</td><td>2</td><td>2.1</td><td>1.05</td><td rowspan="2">3.64</td><td rowspan="2">0.91</td><td>3.18</td><td>1.59</td></tr>
<tr><td>3.75</td><td>1 (chain end groups)</td><td>2</td><td>-</td><td>-</td><td>-</td><td>-</td></tr>
<tr><td>4.07</td><td>3B (CL)</td><td>2</td><td>100</td><td>50</td><td>100</td><td>50</td><td>100</td><td>50</td></tr>
<tr><td>4.18</td><td>3 (CL)</td><td>2</td><td>84.7</td><td>42.35</td><td>26.88</td><td>13.44</td><td>12.27</td><td>6.14</td></tr>
<tr><td>4.6- 4.74</td><td>2,2B,2',2'B (GL)</td><td>4</td><td>84.66</td><td>21.17</td><td>28.6</td><td>7.15</td><td>11.97</td><td>2.99</td></tr>
<tr><td colspan="3">$[CL]/[GL]_{HNMR}$ =</td><td colspan="2">4.36</td><td colspan="2">8.87</td><td colspan="2">18.78</td></tr>
<tr><td colspan="3">$[CL]/[GL]_{Feed}$=</td><td colspan="2">4</td><td colspan="2">10</td><td colspan="2">20</td></tr>
</table>

Table S1. Semi-quantification of the CL: GL ratio among the 3 polyesters: P(CL-co-GL) triol synthesized in the present study

Response	Cell type	Score				
		0	1	2	3	4
**Encapsulation**		0	Partial to complete thin band of granulation tissue/fibrosis surrounding the scaffold	Moderately thick band of granulation tissue/fibrosis surrounding the scaffold	Thick band of granulation tissue/fibrosis surrounding the scaffold	Extensive band of granulation tissue/fibrosis surrounding the scaffold
**Inflammation**	Granulocytes (G) (including neutrophils and eosinophils)	0	Minimal infiltrate, 1-5/HPF	Mild infiltrate, 5-10/HPF	Moderate infiltrate, 10-30/HPF	Heavy infiltrate
	Lymphocytes (L)	0	Minimal infiltrate, 1-5/HPF	Mild infiltrate, 5-10/HPF	Moderate infiltrate	Heavy infiltrate
	Plasma cells (P)	0	Minimal infiltrate, 1-5/HPF	Mmild infiltrate, 5-10/HPF	Moderate infiltrate	Heavy infiltrate
	Macrophages	0	Minimal infiltrate, 1-10/HPF	Mild infiltrate, 10-30/HPF	Moderate infiltrate	Heavy infiltrate
	Multinucleated giant cells (MGCs)	0	Minimal infiltrate, 1-2/HPF	Mild infiltrate, 3-5/HPF	Moderate infiltrate 6-9/HPF	Heavy infiltrate >10/HPF
**Necrosis**	Necrotic debris = nuclear basophilic granular debris and/or cytoplasmic eosinophilic amorphous material surrounding the test material.	0	Minimal amount	Mild amount	Moderate amount	Large amount
**Tissue ingrowth into porous scaffolds (including Total tissue ingrowth adipose tissue, fibrous tissue, mesenchymal tissue)**		0	<25% of Total available space	25-50% of Total available space	50-75% of Available total space	>75% of Available total space

HPF = high power field (field at 400x)

A total inflammation score was also calculated by adding the scores of G+L+P+M+MGCS (range 0-15)

Table S2. Histologic scoring system for Biocompatibility evaluation according to ISO 10993-6:2007, slightly modified to adapt to the present study
